# Pituitary Gangliocytoma Producing TSH and TRH: A Review of “Gangliocytomas of the Sellar Region”

**DOI:** 10.1210/clinem/dgaa474

**Published:** 2020-07-24

**Authors:** Kiyohiko Sakata, Kana Fujimori, Satoru Komaki, Takuya Furuta, Yasuo Sugita, Kenji Ashida, Masatoshi Nomura, Motohiro Morioka

**Affiliations:** 1 Department of Neurosurgery, Kurume University, School of Medicine, Fukuoka, Japan; 2 Department of Pathology, Kurume University, School of Medicine, Fukuoka, Japan; 3 Department of Neuropathology, Neurology Center, St. Mary’s Hospital, Fukuoka, Japan; 4 Division of Endocrinology and Metabolism, Department of Internal Medicine, Kurume University School of Medicine, Fukuoka, Japan

**Keywords:** pituitary gangliocytoma, inappropriate secretion of thyroid-stimulating hormone, thyroid-stimulating hormone, TSH-releasing hormone, mixed gangliocytoma-adenoma, neuroendocrine neoplasm

## Abstract

**Purpose:**

Pituitary gangliocytomas (GCs) are rare neuronal tumors that present with endocrinological disorders, such as acromegaly, amenorrhea-galactorrhea syndrome, and Cushing’s disease. Most pituitary GCs coexist with pituitary adenomas pathologically and are diagnosed as mixed gangliocytoma-adenomas. Herein, we report a case of 45-year-old man who presented with the syndrome of inappropriate secretion of thyroid-stimulating hormone (SITSH) and discuss the pathogenesis of pituitary GCs.

**Methods:**

Pituitary magnetic resonance imaging showed an 8-mm homogeneous and poorly enhanced mass inside the pituitary gland. Endoscopic transsphenoidal surgery was performed under a preoperative diagnosis of thyrotroph adenoma. However, the tumor was finally diagnosed as gangliocytoma without an adenomatous component. The tumor was further analyzed via immunohistochemistry and electron microscopy. Additionally, we searched MEDLINE and PubMed for previously published cases of isolated pituitary GCs and analyzed the reported clinicopathological findings.

**Results:**

The patient showed complete clinical and endocrinological recovery after an operation. The tumor was positive for thyrotropin (TSH), TSH-releasing hormone (TRH), Pit-1, GATA-2, and most neuronal markers. Electron microscopy demonstrated the presence of intracytoplasmic secretory granules and neuronal processes. Co-secreting hypothalamic and pituitary hormone inside the tumor indicated autocrine/paracrine endocrinological stimulation.

**Conclusion:**

Herein, we report a case of SITSH caused by an isolated pituitary gangliocytoma, expressing both TSH and TRH, which, to our best knowledge, is the first reported case of such a condition. The multidirectional differentiation and multihormonal endocrine characteristics of these tumors indicate that they are a member of neuroendocrine neoplasms, further supporting that they are derived from neural crest cells.

Pituitary gangliocytomas (GCs)/mixed gangliocytoma-adenomas (MGAs) have been categorized as neuronal and paraneuronal tumors in the fourth edition of the World Health Organization (WHO) classification of endocrine tumors in 2017 (1). GCs/MGAs are considered rare pathological entities, with only more than 150 cases of pituitary GCs/MGAs reported in the literature. Most lesions occur as mixed tumors with adenomatous and gangliocytic components, rather than isolated GCs, and approximately 75% of the patients exhibit pituitary or hypothalamic hormone hypersecretion (2). Because of the rarity of pituitary GCs, most cases are usually misdiagnosed as pituitary adenomas, as it is difficult to distinguish between the two radiographically before surgery.

Cossu et al recently reviewed 130 cases of pituitary GCs/MGAs, including their case of MGAs (3). Only 19 of these cases (14.6%) were classified as isolated GCs, whereas the others (111 cases, 85.4%) were classified as MGAs. In cases of MGAs, concomitant adenomatous components were identified as mixed somatotroph—lactotroph adenomas (43%), somatotroph adenomas (33%), lactotroph adenomas (14%), and corticotroph adenomas (6%) via immunohistochemistry. Moreover, GCs/MGAs are usually positive for growth hormone–releasing hormone (GHRH), corticotropin-releasing hormone (CRH), GH, and prolactin (PRL) in most cases (3–7). Because of the close relationships between GCs and the pituitary adenomas and the high incidence of endocrinological hypersecretion syndromes, the histogenesis of GCs/MGAs, specifically the derivation of ganglionic cells in the pituitary gland, has been extensively studied (8–11).

We present herein a first case of isolated pituitary GCs that also developed the syndrome of inappropriate secretion of thyroid-stimulating hormone (SITSH), as confirmed by extensive pathological studies. With the latest knowledge, we also reviewed “gangliocytomas of the sellar region” that were previously reported, focusing on their presumed pathogenesis.

## Materials and Methods

### Search strategies of literature review

We searched OVID MEDLINE and PubMed for related articles published before December 2018. The keywords were “gangliocytoma,” “ganglioneuroma,” “neuronal choristoma,” “pituitary,” and “sellar.” Relevant articles in English, German, Russian, and Japanese were also retrieved and reviewed to identify additional papers not detected in the database search. The identified articles were reviewed with a focus on “isolated” pituitary gangliocytomas without an adenomatous component.

### Case presentation and pathological analysis

We describe a unique case of isolated pituitary GC presenting with SITSH. For immunohistochemical analyses, tissues were fixed in 10% formaldehyde and embedded in paraffin. The 5-µm thick sections were stained with hematoxylin and eosin (HE). The remaining serial unstained sections were used for immunohistochemistry. Immunohistochemistry was performed via an immunoperoxidase method with an ENVISION FLEX kit (Agilent/Dako, Tokyo, Japan), using horseradish peroxidase and 3,3’-diaminobenzidine tetrahydrochloride. Protein and endogen peroxidase blockage were performed. The primary antibodies used and their dilution rate are listed in Table 1.

**Table 1.** Primary antibodies used in the immunohistochemistry and their dilution rate

Categories	Antibodies	Clone	Company	Dilution rate
Neuronal/glial markers	Synaptophysin	27G12	Nichirei Bioscience	Prediluted
	Chromogranin-A	DAK-A3	Agilent/Dako	1:400
	Neurofilament	2F11	Agilent/Dako	1:3
	NCAM (CD56)	CD564	Leica Biosystems	1:200
	NeuN	A60	Merk Millipore	1:200
	GFAP	BSR189	Dianova	1:12
Epithelial markers	CAM 5.2	CAM5.2	BD Biosciences	1:15
	CK 5/6	D5/16B4	Agilent/Dako	1:100
	CK 7	OV-TL12/30	Agilent/Dako	1:400
	CK 8	35βH11	Agilent/Dako	1:1200
	CK 20	Ks20.8	Agilent/Dako	1:200
	CK 34βE12	4βE12	Agilent/Dako	1:100
Hormonal markers	GH	Polyclonal	Agilent/Dako	1:4
	PRL	Polyclonal	Agilent/Dako	Prediluted
	TSH	0042	Agilent/Dako	1:2
	ACTH	02A3	Agilent/Dako	Prediluted
	LH	C93	Agilent/Dako	Prediluted
	FSH	C10	Agilent/Dako	Prediluted
	TRH	Polyclonal	BIOSS	1:200
Transcription factors	Pit-1	HPA050624	Sigma-Aldrich	1:2000
	GATA-2	AF2046	R&D systems	1:200
	SF-1	EPR19744	Abcam	1:1000
	Tpit	AMAB91409	Sigma-Aldrich	1:1000
	ER	SP1	Ventana	Prediluted
	TTF-1	8G7G3/1	Agilent/Dako	1:200
Others	Ki-67	MIB-1	Agilent/Dako	1:100
	P53	DO-7	Leica Biosystems	1:200
	CD 3	LN10	Leica Biosystems	1:300
	SSTR2	UMB1	Abcam	1:300
	SSTR5	UMB4	Abcam	1:200

For the ultrastructural study, small fragments of tumor tissue were fixed in 2.5% glutaraldehyde, postfixed in 1.0% osmium tetroxide, dehydrated in graded ethanol, processed through propylene oxide, and embedded in an Epon812. Ultrathin sections stained with uranyl acetate and lead citrate were studied using an H-7650 electron microscope (Hitachi, Tokyo, Japan).

### Review of related literature on isolated GCs in the sellar region

Ganglion cell-containing tumors of the pituitary gland have been reported under various names (4, 6, 11). Several authors have reviewed pituitary GCs, including MGAs, under the former diagnoses of “gangliocytoma,” “ganglioneuroma,” “pituitary adenoma with neuronal choristoma,” “ganglioneuroma and adenoma,” “gangliocytoma with adenoma,” “pituitary adenoma with gangliocytic differentiation,” “adenohypophyseal neuronal choristoma,” and others (2–5, 7, 12).

In 1919, Greenfield first reported a case of a 26-year-old woman with acromegaly with complaints of a headache and visual deterioration. A postmortem examination revealed a huge ganglioneuroma in the sellar region with a maximum size of 9 cm towards the frontal base, middle fossa, and clivus. The neuroma did not infiltrate the brain, but the pituitary gland could not be found (13). Citing this case later in 1930, Courville suggested that the ganglioneuroma might have originated in the tuber cinereum (14). Alpes and Grant also reported a case of a 16-year-old man who presented with visual deterioration and feminization. This patient was found to have a large intra- and suprasellar tumor that extended up to the third ventricle that was diagnosed as a ganglioneuroma following consequent transcranial surgery and histopathological examination (15). Some cases before the advent of computed tomography (CT) and magnetic resonance imaging (MRI), including the ones discussed here, were reported to have a large extrasellar component, although their hypothalamic origin could not be ruled out (13–16). Moreover, in those days, radiation therapy was acceptable as the first-line therapy, which probably caused difficulty for obtaining an accurate pathological diagnosis because of the difference in sensitivity to radiation (17, 18).

In 1929, Kiyono reported a case of a true intrasellar tumor with a gangliocytic component detected via postmortem examination in a 59-year-old woman who had died of pulmonary tuberculosis. In his description, the gangliocytic component with 2 or multiple nuclei was found in the central part of the tumor that was predominantly composed of adenomatous cells, making it the first reported case of pituitary MGA (19). Benda briefly reported a case of a 6 × 11 mm pituitary posterior lobe tumor consisting of numerous large to small neurons, identified via postmortem examination of a 72-year-old woman (20). This was again reported by Casper 6 years later (21) and might have been the first case of a pituitary GC.

After excluding the typical MGAs, we included 32 cases of GCs in the sellar region in the review. Of these, 11 cases were “ganglioneuromas” (assumed to be of sellar origin without immunohistochemical studies) (Table 2), and 21 cases were GCs without an adenomatous component in the sellar region diagnosed by immunohistochemistry (Table 3) (5, 13–16, 18, 20–37). In this review, we avoid the use of “pituitary” GCs because the precise relationship between the tumor and the pituitary gland inside the sella had not been described for some of the cases (13–16, 29, 34). As shown in Tables 2 and 3, middle-aged women (mean age, 43.1 years; female/male, 23/9) were predisposed to develop GCs in the sellar region. Endocrinological symptoms of pituitary hypersecretion were seen in 65.6% (21/32) of all cases and 76.2% (16/21) of the recent series. These symptoms included acromegaly (8 cases), amenorrhea-galactorrhea (6 cases), Cushing’s syndrome (6 cases), and SITSH (1 case [our presented case]). The majority of these 32 cases, that spanned over a century, lacked immunohistochemical evaluation. Of the recent cases, 10 were evaluated for hypothalamic hormones, and 8 of them (80%) demonstrated immunoreactivity to GHRH, CRH, or TSH-releasing hormone (TRH). Additionally, among these 10 recent cases, 4 (40%) demonstrated immunoreactivity to pituitary hormones. In some cases, the tumor cells exhibited overlapping immunoreactivity to the same hypothalamic–pituitary hormonal axis (35, 37). Most of these pathological findings correspond to the endocrinological symptoms of the respective cases.

**Table 2.** Review of 11 cases of ganglioneuromas in the sellar region diagnosed before immunohistochemistry

Author (Ref No.)	Year	Age/Sex	Endocrinological Symptoms	Visual Symptoms	Locations	Treatment/Outcome Comment
Greenfield (13); Courville (14)	1919; 1930;	26/F	Acromegaly	Yes	Huge skull base	Patient died a few days after TCO
Alpers and Grant (15)	1931	16/M	Feminization	Yes	IS/SS/IIIrd	Patient died 1 day after TCO
Benda and Casper (20, 21)	1933	72/F	Not described	No	IS*	Autopsy (died by pulmonary disease)
Robertson et al (22); Serebrin (23)	1964; 1984	45/F	Dysmenorrhea	No	IS Sphenoid sinus	Unrelated death 20 years after TCO; autopsy revealed recurrent tumor
Jakumeit et al (24)	1974	41/F	Amenorrhea	Yes	IS/SS	Treated via TSO, total removal
		37/F	Cushing	No	IS/Sphenoid	Treated via TSO, total removal
		56/M	Acromegaly	Yes	IS/SS	Treated via TCO, total removal
Arseni et al (16)	1975	5/F	Diabetes insipidus	Yes	IS/SS/IIIrd	Treated via TCO and RT
		7/M	Hypothyroidism	Yes	IS/SS	Treated via TCO
Ule and Waidelich (25)	1976	65/F	None	No	IS	Autopsy (died by thyroid carcinoma)
Nikonov (26)	1981	52/F	Acromegaly	Yes	IS/SS/CS	RT→ Patient died 5 days after TCO

Abbreviations: CS, cavernous sinus; F, female; IIIrd, third ventricle; IS, intrasellar; M, male; RT, radiation therapy; SS, suprasellar; TCO, transcranial operation; TSO, transsphenoidal operation.

**Table 3.** Review of 21 cases of gangliocytomas in the sellar region diagnosed by immunohistochemistry

						Immunohistochemical Results of GC	
Author (Ref No.)	Year	Age/Sex	Endocrinological Symptoms	Visual Symptoms	Locations	Pituitary Hormone	Hypothalamic Hormone
Asa et al (27)^*a*^	1984	62/F	Acromegaly	Yes	IS/SS	All negative	GHRH (+)
Asa et al (28)	1984	58/F	Cushing	No	IS	With ACTH-AHCH(+)	CRH (+)
Nishio et al (29)	1987	58/F	Cushing	No	IS	Not described	CRH, SST, OXT (+)
Yamada et al (30)	1990	47/F	None	No	IS/SS	All negative	SST (+)
Baysefer et al (31)	1997	35/M	Cushing	No	IS	Not described	Not described
Saeger et al (32)	1997	34/F	Acromegaly	Not described		All negative	GHRH (+)
McCowen et al (18)	1999	36/M	Hyper-PRL	Yes	IS/SS	PRL (+)	Not described
Geddes et al (33)	2000	53/F	Acromegaly	No	IS/SS	Not described	Not described
		54/M	Cushing	No	IS^*b*^	With ACTH-BI (+)	CRH negative
Ishidro et al (34)	2005	66/F	Acromegaly	No	Parasellar	All negative	GHRH (+)
Qiao et al (5)	2014	Mean 34.3 y; 5/F, 2/M	None: 3 Hyper-PRL: 3 Hyper-GH,PRL: 1	Not described			
Domingue et al (35)	2015	62/F	Cushing	No	IS/sphenoid	ACTH (+)	CRH (+)
Petrakakis et al (36)	2016	49/F	Hyper-PRL	Yes	IS	Not described	Not described
Donadille et al (37)	2017	59/F	None	Yes	IS/SS	GH (+) ACTH (+)	GHRH (+)
Present case	2020	45/M	SITSH	No	IS	TSH (+)	TRH (+)

Abbreviations: AHCH, adenohypophyseal cell hyperplasia; BI, basophil invasion; F, female; GC, gangliocytic cell; IS, intrasellar; M, male; OXT, oxytocin; SS, suprasellar; SST, somatostatin; y, years-old.

^*a*^This patient underwent radiation therapy for acromegaly 25 years ago.

^*b*^The tumor was located at the posterior pituitary.

## Case Report

### Presentation and examination

A 45-year-old obese man (height, 178.7 cm; weight, 97 Kg; body mass index, 30.8 kg/m^2^) complained of general fatigue and drowsiness at work. A blood examination revealed severe diabetes mellitus (HbA1c 10.6%), and he was accordingly referred to our university. Treatment for diabetes mellitus was started and extensive evaluations for sleep apnea syndrome were performed. Although treatment using a continuous positive airway pressure mask was initiated, general fatigue continued. Concurrently, SITSH was diagnosed based on the following findings: serum TSH, 6.890 µIU/mL; free T3, 4.9 pg/mL; and free T4, 2.29 ng/dL.

**Figure 1.**
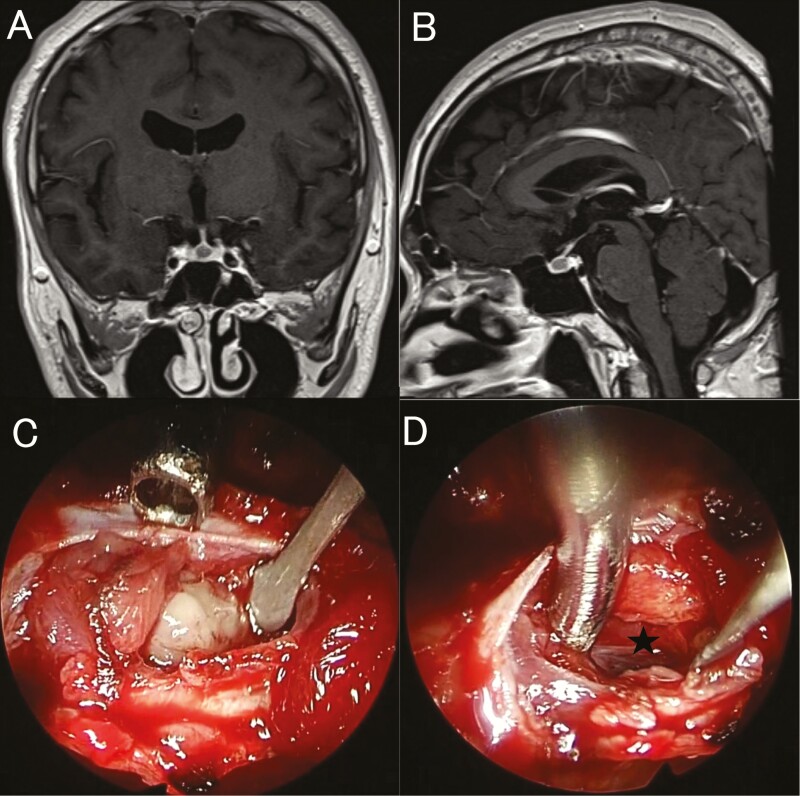
Magnetic resonance imaging (MRI). Preoperative contrast-enhanced coronal (**A**) and sagittal (**B**) MRIs showing a 5 × 6 × 8 mm less-enhanced mass lesion inside the pituitary gland. Intraoperative images show a well-circumscribed whitish tumor after splitting the pituitary gland (**C**), which was completely removed via a fine dissection plane. **D:** The arachnoid membrane can be seen over the resection cavity (star).

Magnetic resonance imaging of the pituitary gland revealed a poorly enhanced mass measuring 5 × 6 × 8 mm (Fig. 1A and 1B). The TRH loading test showed a low and delayed TSH response (pre-TSH, 6.89 µIU/mL; max TSH, 10.8 µIU/mL; 60 minutes after TRH loading). However, there were no abnormal responses for both GH and PRL on several other loading tests. The absence of a family history of SITSH or TRβ gene mutations prompted the diagnosis of thyrotroph adenoma.

Initial treatment with the somatostatin analog (SSA) did not yield any response. Further, the free T4 levels remained over 2 ng/dL after 3 courses of lanreotide autogel (90 mg). Since his diabetes mellitus was already under control, we decided to remove the tumor surgically.

### Operative findings and postoperative course

The surgery was performed using the standard endoscopic endonasal transsphenoidal approach. The pituitary gland appeared normal on the surface. However, a midline split revealed a well-circumscribed whitish tumor inside the pituitary gland (Fig. 1C). Complete tumor resection was achieved (Fig. 1D), and tumor tissues were collected wherever possible. As the intraoperative pathological diagnosis ruled out a pituitary adenoma, tissue samples for electron microscopy were obtained.

His serum TSH levels decreased drastically to 0.320 µIU/mL on postoperative day 1. However, the patient developed transient diabetes insipidus that was treated with oral desmopressin acetate tablets. Three days after the operation, he was diagnosed with influenza type A, followed by hyponatremia with a minimum serum Na level of 118 mEq/L. Electrolyte levels were closely monitored during the treatment for influenza, and he was discharged 18 days after the operation without any electrolyte management. Oral administration of hydrocortisone and levothyroxine was started after the operation, which was tapered and ceased completely after 3 months.

 The patient’s complaint of fatigue improved, and his cardiac heart rate was reduced by 10 bpm. Blood examination 6 months after the operation revealed an improvement in endocrinological parameters, including serum TSH (0.808 µIU/mL), free T3 (2.9 pg/mL), and free T4 (1.35 ng/dL). The TRH loading test showed a normal TSH response (pre-TSH, 0.589 µIU/mL; max TSH, 7.830 µIU/mL) 30 minutes after TRH loading. The patient has remained healthy for 2 years without tumor recurrence.

### Pathological and radiographical findings

**Figure 2.**
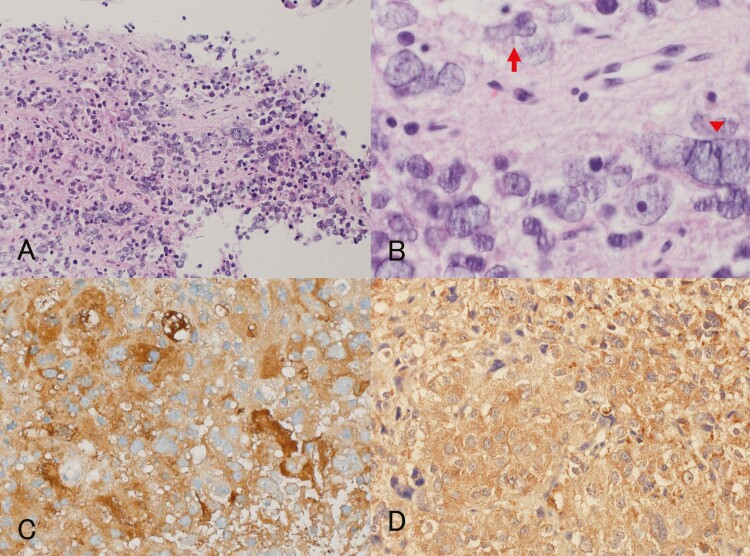
Hematoxylin and eosin staining shows tumor cells with small to large bizarre nuclei against a background of finely, fibrillar, neuropil-like matrix (magnification ×100) (**A**). **B:** Individual tumor cells with irregular shapes and dysplastic nuclei containing a prominent nucleolus can be seen (magnification ×400). Some cells display cleaved nuclei (arrow), and some are multinucleated (arrow head). Immunohistochemical staining for pituitary and hypothalamic hormones revealed a diffuse co-expression of both TSH (**C**) and TRH (**D**) for the cytoplasm of tumor cells (magnification ×200).

**Figure 3.**
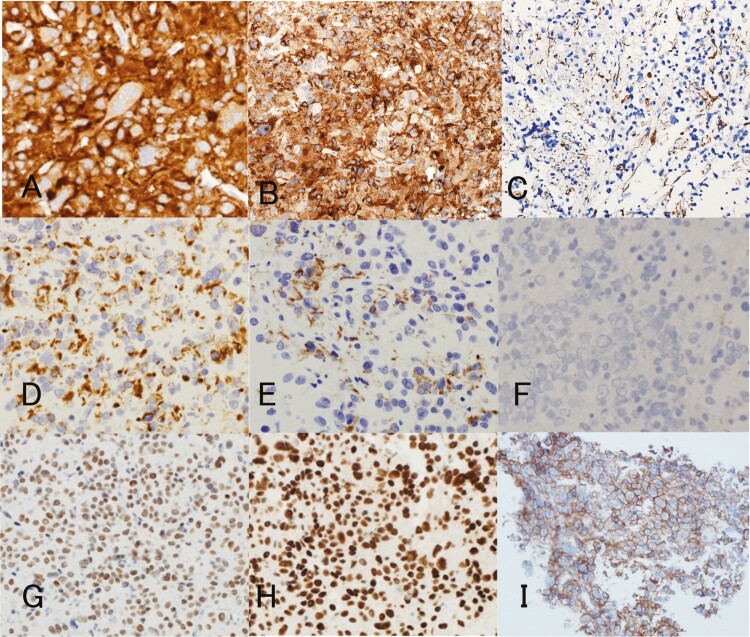
Immunohistochemical study reveals strong reactivity for neuronal markers such as synaptophysin (**A**), chromogranin A (**B**), and neurofilament (**C**). Scattered expression of cytokeratins for cytoplasm of the tumor cells are revealed using CAM 5.2 (**D**) and CK7 (**E**). Negative for GFAP rules out ganglioglioma (**F**). Immunostaining for the transcription factor Pit-1 (**G**) and GATA-2 (**H**) display strong and diffuse nuclear staining in the whole tumor cell component. The cytomembranes of the tumor cells stained positively for SSTR2A (**I**), which is 1 of the 5 subtype receptor families for the ligand somatostatin (magnification ×200 [**A**, **B**, **D–F**], and magnification ×100 [**C**, **G–I**]).

Postoperative MRI revealed complete tumor resection. Hematoxylin and eosin staining showed that the tumor was composed of small- to large-sized neuronal or ganglionic cells containing abundant acidophilic cytoplasm and nuclei with a prominent nucleolus against a background of fine, fibrillar, neuropil-like matrix (Fig. 2A and 2B), which were diffusely immunoreactive to synaptophysin, chromogranin A, neurofilament, and NCAM (CD56), and partially immunoreactive to NeuN (Fig. 3A–3C). Interjacent small cells were considered to be reactive lymphocytes and not adenomatous cells based on their immunoreactivity to CD3. The cytoplasm of the tumor cells with peripheral displacement of the nucleus was immunoreactive for low-molecular-weight keratins, CAM 5.2, and patchy reactive for CK7 (Fig. 3D and 3E), but not for CK5/6, CK 8, CK 20, and CK 34βE12, supporting the exclusion of paragangliomas. Further, the absence of immunoreactivity to glial fibrillary acidic protein confirmed the exclusion of ganglioglioma (Fig. 3F). Although Ki-67 staining revealed a labeling index of 2.6%, no other atypical features were detected. Only 0.4% of the entire tumor showed P53 immunopositivity. Based on these findings, a final pathological diagnosis of isolated GC was made.

With respect to the pituitary and hypothalamic hormones, the cytoplasm of the tumor cells showed diffuse but strong immunoreactivity for TSH (Fig. 2C) and equivocal faint positivity for gonadotropin; however, it was negative for GH, PRL, and ACTH. Meanwhile, it also showed diffuse but strong immunoreactivity to TRH (Fig. 2D). Transcription factors such as the acidophilic cell lineage transcription factor Pit-1, SF-1, Tpit, ER, and GATA-2 are important to pituitary cytodifferentiation from the Rathke pouch stem cell. Further immunohistochemical studies for these transcription factors revealed diffuse and strong nuclear immunoreactivity for Pit-1 and GATA-2 (Fig. 3G and 3H), but not for SF-1, Tpit, and ER, confirming thyrotropic cell differentiation of tumor cells. Thyroid transcription factor-1 (TTF-1) is a tissue-specific transcription factor that regulates the expression of selected genes in the thyroid, lung, and diencephalon for embryonic development and differentiation; it is well known that it aids in nuclear expression, specifically that of posterior pituitary, thyroid, and lung tumors (1, 38). All tumor cells stained negatively for TTF-1. Somatostatin receptor (SSTR) status may predict treatment response to first- and second-generation SSAs. With respect to SSTR membranous immunopositivity proposed by Volante et al (39), more than 50% of tumor cells stained positively for SSTR2 (Score 3 (Fig. 3I)); however, none of the cells stained positively for SSTR5 (Score 0).

**Figure 4.**
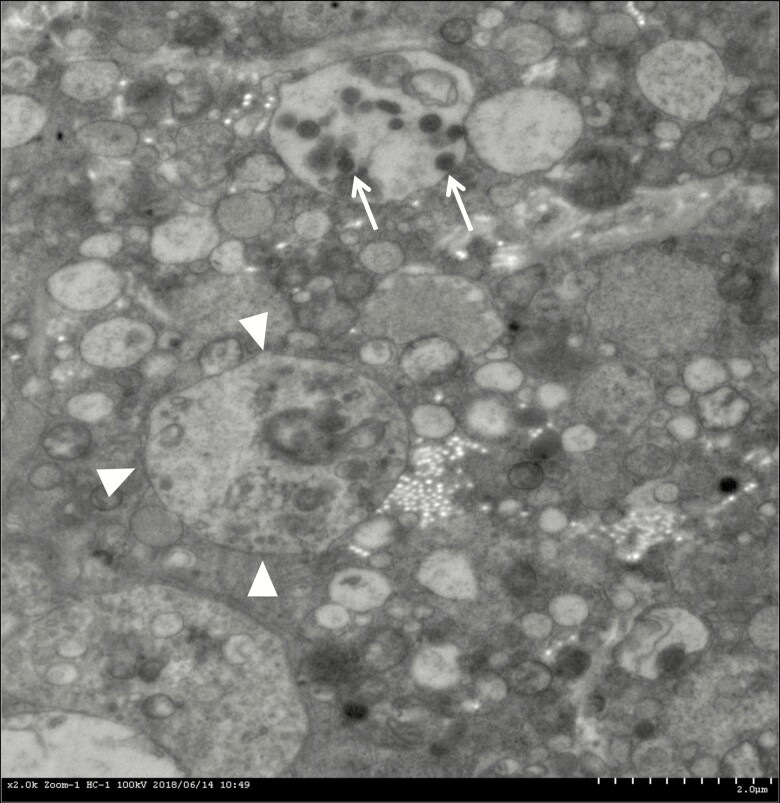
Electron microscopy revealed that the tumor cell has a light nucleus with a prominent nucleolus (arrow head), which are surrounded by many secretary granules, synaptic vesicles, and some lysosomes in the cytoplasm. Typical neuronal process contains both dense core vesicles (white arrow) and clear vesicles.

Based on these immunohistochemical findings, this tumor was considered to be differentiated into an entirely neuronal lineage rather than mixed or interjacent with 2 components. Electron microscopy revealed the tumor cell has a light nucleus with a prominent nucleolus, which is surrounded by many secretary granules, synaptic vesicles, and some lysosomes in the cytoplasm. Typical neuronal processes contained both dense core vesicles and clear vesicles (Fig. 4).

## Discussion

### Incidence of pituitary GCs/MGAs and hypersecretion of TSH

The origin and histogenesis of pituitary gangliocytic tumors have remained an enigma. Gangliocytomas have the potential to manifest anywhere within the central nervous system, but it is well known that the majority of pituitary gangliocytic tumors occur in combination with pituitary adenomas. However, pituitary gangliocytic tumors are rare, accounting for only 0.14%–1.42% of all sellar lesions (2, 9, 10). Therefore, isolated GCs are considered rather as cases that could not be identified as having an adenomatous component in microscopic and immunohistochemical studies (6). Meanwhile, gangliocytic cells may secrete pituitary or hypothalamic hormones, thus directly manifesting syndromes of pituitary hypersecretion such as acromegaly, Cushing’s disease, and amenorrhea-galactorrhea syndrome (18, 27–29, 32, 34–37).

In 1960, Jailer and Holub first reported a case of a patient with symptoms of hyperthyroidism and expanded sella on X-ray imaging. They called this condition SITSH, which was presumably due to a pituitary adenoma (40). Since then, more than 450 cases of thyrotroph adenomas have been published. These adenomas account for 0.5%–3% of all pituitary tumors, and their age-standardized national incidence is 0.15 per 1 million population annually (41). Although the incidence of SITSH is low, it has neither been reported with isolated GCs nor with MGAs. The majority of functioning thyrotroph adenomas and somatotroph adenomas are sensitive to SSA treatment, correlating with expression of SSTR2 and SSTR5 (42). In the current study, the patient did not respond to preoperative SSA treatment, and the reason is unknown. However, in thyrotroph adenomas, it has been reported that the presence of SSTR5 is correlated with both in vivo and in vitro response to SSA (43, 44). In addition, the intracellular downstream mechanisms after SSTRs, including the tumor microenvironment, may be different between thyrotroph adenomas and this tumor (45, 46).

### Pathogenesis of GC/MGA development

The simultaneous occurrence of GCs and pituitary adenomas at high rates is unlikely to be a simple coincidental finding and is more likely to involve a causative relationship between the 2 lesions, which needs to be understood. The pituitary gland is composed of the anterior, intermediate, and posterior lobes. The first 2 are derived from Rathke’s pouch, an invagination of the oral ectoderm, whereas the last is derived from the overlying diencephalic neural ectoderm.

Immunohistochemical studies of GCs/MGAs have led to several hypotheses explaining the pathogenesis of these tumors (2–4, 6, 8–12, 27, 47). First, abnormal migration of hypothalamic neurons within the adenohypophyseal parenchyma may occur during embryogenesis (47). Secretion of hormones by the ganglionic cells of hypothalamic origin could lead to the development of adenomas. However, this hypothesis was contradicted because of a lack of correlation between the released hypothalamic hormones and the adenoma cell secretions in some cases (12, 27). Moreover, the hypothalamic-releasing hormones secreted by the ganglionic cells seem to promote proliferation and not initiate tumorigenesis of pituitary adenomas.

Second is the widely accepted theory that pre-existing adenoma cells transdifferentiate into gangliocytic cells with the acquisition of neural differentiation (2, 8). Ultrastructural studies by Horvath et al showed the presence of transitional cell forms between neurons and adenohypophyseal cells (8). Additionally, there were some clinical cases that exhibited neuronal components inside adenomas that could be denominated as neuronal metaplasia (48–51). Although it may be impossible for neoplastic pituitary cells to transform into well-differentiated mature neuronal tumors with the dominating embryological concepts (9), “metaplasia” may arise not only as a result of proliferation and transformation of immature cells, but also of the transformation of mature cells under various conditions such as sublethal injury, chronic inflammation, and vitamin A deficiency (52). However, because a pre-existing adenoma is an absolute condition, it is difficult to explain the occurrence of isolated GCs or other neuronal tumors, including neurocytomas and neuroblastomas (1).

Third is an alternative theory advocated by Kontogeorgos et al that stipulates that neuronal and adenohypophyseal cells may derive from a common pituitary stem/progenitor cell (9, 53). Transdifferentiation of adenoma cells into neurons is yet to be established, but adenoma cells are known to express some neuronal epitopes, particularly synaptophysin, and may, therefore, have the potential to transdifferentiate (51, 54). Some studies have demonstrated that neuronal markers, including NeuN and neurofilament, are expressed in the adenomatous cell component in MGAs, suggesting a common origin for the gangliocytic and adenomatous cells (9, 10, 51, 55). This hypothesis can explain both the differences in the hormonal profiles between adenomatous and gangliocytic components as well as the development of isolated GCs and MGAs.

However, not all gangliocytic tumors within a sellar lesion follow the same pathobiological process, as can be seen from 2 cases of isolated posterior pituitary GCs that did not associate to the adenohypophyseal cells (21, 33). Meanwhile, Horvath et al have demonstrated that the ganglionic cells in the posterior pituitary could be the result of the ectopic migration of neuronal cells (47), and these migrated cells could give rise to such tumors.

### Stem cell theory and transcription factors in pituitary GCs/MGAs

It is interesting to note that although the tumor did not have an adenomatous component, the present case had comparable specific-lineage transcription factors to that of thyrotroph adenomas such as Pit-1 and GATA-2; this case also exhibited specific neuronal differentiation. Several cell-restricted transcription factors such as PROP1, Pit-1, SF-1, Tpit, ER, and GATA-2 during cytodifferentiation from the pituitary stem cells (PSCs) have been identified, and PSCs can generate each differentiated cell within pituitary tissues to support organ repair and regeneration. However, it is still unknown whether PSCs are a single population with multipotent differentiation capacity or distinct populations with a more restricted lineage commitment (56–58). Alternatively, a theory has been proposed that tumor stem cells (TSCs) arising from tissue-specific stem cells following an oncogenic transformation may initiate the tumorigenesis and possess stem cell characteristics of self-renewal, maintenance, and the potential to differentiate and grow into a heterogeneous tumor (57–59). Many studies have shown the presence of populations of undifferentiated cells with clonogenic capability within pituitary tumors, suggesting that the TSC model may be relevant to pituitary neoplasms (57). Chen et al found that isolated cells that expressed neural stem/progenitor cell markers formed neurospheres in vitro and generated daughter cells with the capability to differentiate into multiple neural lineages (60). However, they failed to demonstrate the production of pituitary hormone cell types, which may be derived from neural crest stem cells and form pericytes associated with the pituitary vasculature (58, 61).

Some authors have reported that the acidophilic lineage transcription factor Pit-1 is expressed in both adenomatous and gangliocytic cells in MGAs and speculated a common ectodermal origin for the 2 components or transdifferentiation of neuroendocrine cells to a more neuronal phenotype (55, 62). The more likely scenario involves transdifferentiation or differentiation from a progenitor cell originating in the neuroendocrine component that has common transcription factors, rather than neuronal differentiation from well-differentiated adenoma cells, and may generate MGAs and other neuronal tumors in the sellar region.

### Proposal for categorization as neuroendocrine neoplasms

A new classification of neoplasms of adenohypophyseal cells proposed to rename them to “pituitary neuroendocrine tumors (PitNETs)” for comprehensive cognizance of their wide pathological spectrum and clinical behavior (63, 64). However, the 2017 WHO classification categorized pituitary tumors according to pituitary cytogenetic lineage and morphofunctional subtypes (1, 64). Concurrently, the WHO International Agency for Research on Cancer has also classified neuroendocrine neoplasms (NENs) across different organ systems, including PitNETs, which have been previously classified using site-specific terminologies and chaotic criteria under a common framework (65).

Although it has long been debated about their triploblastic origin (66), neuroendocrine cells that have acquired neuroendocrine integration from various stem/progenitor cells are found in almost every individual’s organs and are known to form the diffused neuroendocrine system. Neuroendocrine neoplasms express a variable spectrum of proteins shared with their normal cell counterparts at specific anatomical locations, including markers of general neuroendocrine differentiation as well as site-specific markers such as hormones and transcription factors (65, 67).

Furthermore, these arguments concerning the pathogenesis of mixed tumors are also seen in adrenal or gastroenteropancreatic NENs (68–71). “Mixed neuroendocrine–non-NENs (MiNENs)” are defined by the association of at least 2 morphologically different neoplastic components, with the requirement that the minor component comprise ≥ 30% of the tumor, which have been introduced in the fifth edition of the WHO classification of the digestive system tumors published in 2019 (69, 70, 72). Many NENs may show evidence of non-neuroendocrine differentiation, whereas many non-neuroendocrine tumors may contain subpopulations of neuroendocrine cells. These phenomena, including multidirectional differentiation and multihormonal endocrine characteristics, have been observed in NENs of undisputed neural crest origin and in tumors of other origin (73).

The clinical implication of cytokeratin expression in the cytoplasm of gangliocytic cells in MGAs, which has been considered evidence of transdifferentiation of adenoma epithelial cells into gangliocytic cells, is still debated to date (6, 8, 9, 11). However, some authors implied a participation of neural crest-derived cells (9, 11). The immunohistochemical results of positivity for CAM5.2 and CK7 but negativity for CK20 were confirmed in a subpopulation of NENs as well as that of pituitary adenomas, which may indicate organ-related differentiation in neoplasms (38, 74). While the European Pituitary Pathology Group has proposed the diagnostic algorithms for PitNETs, it is interesting to note that the pathological features of GCs/MGAs are more similar to those of NENs than to those of functional adenomas (67, 75).

### Pituitary tumors co-secreting both hypothalamic and pituitary hormones

The phenomena in pituitary tumors exhibiting co-secretion of hypothalamic upstream hormones and pituitary downstream hormones in the same tumor cell have been reported on rare occasions. In acromegaly, co-secreting GH-GHRH-producing adenoma has been reported in cases of both pure pituitary adenoma and adenomatous components in MGA (76, 77). Moreover, some investigations have demonstrated that GHRH can synthesize not only in the normal pituitary gland, but also concurrently in somatotroph adenoma cells (78, 79).

In addition, co-secretion of both CRH and ACTH in the same tumor cell of both pure pituitary adenoma and isolated GC have also been reported in patients with Cushing’s disease (35, 80). Domingue et al suggested that these tumor cells can induce adrenal hypersecretion both directly thorough ACTH secretion and indirectly via paracrine stimulation of the normal corticotroph cells (35). Asa et al also reported corticotroph hyperplasia of the surrounding pituitary cells but not corticotroph adenomas in CRH-producing GC (28), supporting the autocrine/paracrine mechanisms. It is interesting that tumors exhibiting co-secretion of both CRH and ACTH are occasionally seen in gastroenteropancreatic NENs rather than in pituitary tumors (81–83).

 In our case, diffuse co-secretion of both TSH and TRH was seen in the tumor cells, which is an undisputed cause of SITSH. In general, most thyrotroph adenomas are reported to be large and invasive at diagnosis, and about 30% of the TSH-immunopositive adenomas are clinically silent (41, 44, 84). Moreover, 2 cases of MGAs wherein the gangliocytic component exhibited TSH immunoreactivity have been reported; however, these patients did not clinically manifest hyperthyroidism or SITSH (6, 85). Although our case resembled a microadenoma, the SITSH may have resulted in continuous activation across the hypothalamic–pituitary–thyroid axis. More specifically, autocrine/paracrine stimulation can occur inside and/or outside the tumor cells. Some studies have suggested that the TRH gene is expressed not only in tumoral anterior pituitary cells, but also in normal anterior pituitary cells. Further, TRH signaling induces TSHβ expression through GATA-2 as a principal mediator, which may act as an autocrine/paracrine regulator of TSH secretion (86–90).

### Study strengths and limitations

The features of multipotential cells in pituitary GCs/MGAs that lead to mature neuronal differentiation or to hypothalamic–pituitary axis hormonal synthesis could be explained by concepts of systemic NENs. Importantly, it may support the theory that pituitary GCs/MGAs arise from common stem/progenitor cells, specifically from specialized neuroendocrine cells derived from the neural crest in the embryo. Interestingly, some investigators have recently demonstrated that the mesenchyme on the rostral side of Rathke’s pouch originates from the neural crest. Moreover, neural crest cells invade the embryonic anterior pituitary, which is believed to derive solely from the adenohypophyseal placode in a stepwise manner and differentiate into all hormone-producing cell lineages and vascular pericytes (91–94). The presence of such stem/progenitor cells can be explained by the pathogenesis of GCs/MGAs, including wide-ranging differentiation from a subpopulation of pure pituitary adenomas to pure gangliocytomas, including these mixed tumors with various proportions.

In this literature review, only 21 cases of GCs in the sellar region with immunohistochemical studies were identified during the past 35 years, thus limiting the generalizability of our findings. Further research and collection of data on these tumors, including MGAs, are necessary.

## Conclusions

We report herein a rare case of pituitary GC-manifesting SITSH. Immunohistochemical studies revealed diffuse co-secretion of both TRH and TSH by the tumor, with diffuse expression of lineage-restricted transcription factors (ie, Pit-1 and GATA-2), which may have resulted in a continuous autocrine/paracrine regulation of TSH secretion. The high frequency of co-occurrence of gangliocytic and adenomatous components within a neuroendocrine tumor suggests that they arise from multipotent progenitor cells that can differentiate into multidirectional morphology and have the capacity for multihormonal synthesis; these abovementioned aspects result in syndromes of pituitary hypersecretion in an autocrine/paracrine manner.

## Data Availability

The datasets and analyzed data in this current study are available from the corresponding author upon reasonable request.

## Abbreviations

GC: gangliocytoma
MGA: mixed gangliocytoma-adenoma
NET: neuroendocrine tumors
PitNETs: pituitary neuroendocrine tumor
PRL: prolactin
PSC: pituitary stem cell
SITSH: syndrome of inappropriate secretion of thyroid-stimulating hormone
SSA: somatostatin analog
SSTR: somatostatin receptor
TSC: tumor stem cell
WHO: World Health Organization

## Additional Information

***Disclosure Summary:*** The authors have no personal financial and institutional interest in any of the drugs, materials, or devices described in this article.
